# Evaluation of vitamin D levels and biochemical markers in infants diagnosed with laryngomalacia

**DOI:** 10.55730/1300-0144.5707

**Published:** 2023-04-27

**Authors:** Nagihan BİLAL, Ömer Faruk ÇINAR, Sevcan İPEK, Muhammed SEYİTHANOĞLU, Adem DOĞANER, Muhammed Gazi YILDIZ

**Affiliations:** 1 Department of Otorhinolaryngology, Faculty of Medicine, Kahramanmaraş Sütçü İmam University, Kahramanmaraş, Turkiye; 2 Department of Pediatric Critical Care, Faculty of Medicine, Kahramanmaraş Sütçü İmam University, Kahramanmaraş, Turkiye; 3 Department of Biochemistry, Faculty of Medicine, Kahramanmaraş Sütçü İmam University, Kahramanmaraş, Turkiye; 4 Department of Biostatistics and Medical Informatics, Faculty of Medicine, Kahramanmaraş Sütçü İmam University, Kahramanmaraş, Turkiye

**Keywords:** Laryngomalacia, vitamin D deficiency, calcium, parathormone, fiberoptic laryngoscope

## Abstract

**Background/aim:**

The pathology of laryngomalacia is still not clear. The aim of this study was to investigate the relationship between vitamin D levels and laryngomalacia, and to evaluate vitamin D levels according to the classification of laryngomalacia.

**Materials and methods:**

This retrospective study was conducted in the Kahramanmara**ş** S**ütçü İ**mam University Medicine Faculty’s Otorhinolaryngology Clinic between June 2014 and January 2021. Laryngomalacia was classified. Laboratory tests for all patients included calcium (Ca), phosphorus (P), parathormone (PTH), blood urea nitrogen (BUN), creatinine (Cre), alanine transaminase (ALT), and 25-hydroxy vitamin D (25-OH-D).

**Results:**

Evaluations were performed for 64 infants with laryngomalacia, including 41 male and 23 female infants with a mean age of 4.6 ± 3.0 months, and a control group of 64 healthy infants with a mean age of 4.5 ± 2.8 months. A statistically significant difference was determined between the laryngomalacia group and the control group with respect to 25-OH-D and PTH levels (p < 0.001). When data were examined according to laryngomalacia types, a statistically significant difference was determined between the groups for 25-OH-D, Ca, P, PTH, and ALT values. The 25-OH-D level was statistically significantly lower in the severe laryngomalacia group than in the mild and control groups (p < 0.001). A statistically significant difference was determined between the moderate and severe laryngomalacia groups and the control group regarding PTH levels (p < 0.001).

**Conclusion:**

Vitamin D deficiency may have a role in the etiology of laryngomalacia, and this view is supported by the finding that there was a decrease in vitamin D levels associated with laryngomalacia classification. In addition, the reduction in PTH levels in infants with laryngomalacia may be explained by the change in Ca metabolism. It would be appropriate for further studies to investigate the response to vitamin D replacement therapy in patients with moderate and severe laryngomalacia.

## 1. Introduction

Maternal vitamin D deficiency is a common public health problem [1,2]. The role of maternal nutritional status in normal fetal development has stimulated great interest among researchers. Many reports worldwide have shown a high prevalence of vitamin D deficiency [3,4]. In pregnant women, vitamin D passes from the placenta to the fetus and the mother is therefore the only source of vitamin D substrate for the developing fetus. Maternal vitamin D deficiency is defined as a serum 25-hydroxyvitamin D (25-OH-D) level of <50 nmol/L and has been shown to be associated with vitamin D deficiency in the fetus [5].

Laryngomalacia is the most commonly seen congenital laryngeal anomaly in children, constituting 75% of all infantile stridor cases [2,6]. It is characterized by inspiratory stridor because of supraglottic collapse. The severity of the respiratory problem increases while feeding, lying on the back, and crying. Generally symptoms start to be seen within the weeks after birth, and while there is clinical worsening around the fourth month, it is seen to spontaneously completely recover in the second year [7,8]. Diagnosis is based on the observation of supraglottic collapse in flexible laryngoscope examination [9]. Although most cases do not require surgery, intervention may be required when there is severe respiratory failure and the infant is not developing [9,10].

The classic effects of vitamin D deficiency in pregnancy and neonates have included late hypocalcemia and nutritional rickets. Nevertheless, recent studies have linked vitamin D to fertility and 25-OH-D with several clinical conditions in pregnancy including preeclampsia, gestational diabetes, higher incidence of cesarean section, and preterm birth, while in infants, the clinical conditions are low birth weight, lower bone mass, and possible relationships with the development of diseases such as bronchiolitis, asthma, type 1 diabetes, multiple sclerosis, and autism among the nonclassical actions of vitamin D [11]. However, many of these associations have been inconsistently reported in the literature despite extensive research into vitamin D in the context of pregnancy. Furthermore, the lack of clarity regarding optimal vitamin D levels in relation to pregnancy outcomes has led to inconsistencies in the guidelines for classifying vitamin D deficiency or defining the level of supplementation required to support a healthy pregnancy [12]. The role of vitamin D in calcium and phosphorus regulation for healthy bone mineralization is well known. Increasing evidence has raised interest in the role of vitamin D in many extraskeletal functions, including inflammation and immunoregulation [13].

The physiopathology of laryngomalacia has not yet been fully clarified. Theories have been proposed related to neurological pathologies caused by submucosal nerve hypertrophy or changes in the sensorimotor integrative function [8]. Recent studies have shown that vitamin D may have a role in the etiology of laryngomalacia [2,8]. With the goal of contributing to these studies in the literature, the aim of this study was to evaluate vitamin D levels according to the classification of laryngomalacia.

## 2. Materials and methods

This study was conducted in accordance with the principles of good clinical practice and the Declaration of Helsinki. Approval for the study was granted by the local ethics committee (session no. 11, dated 27.01.2021). This retrospective study was conducted in the Kahramanmaraş Süt**çü İ**mam University Medicine Faculty’s Otorhinolaryngology Clinic between June 2014 and January 2021.

The study group included 64 infants (41 male and 23 female) aged <1 year with no syndromes and no congenital anomalies who were diagnosed with laryngomalacia in flexible endoscopic examinations. Infants were excluded from the study if they had any congenital respiratory pathology (subglottic hemangioma, laryngeal web, and stenosis), acquired stridor, a history of intubation, presence of infection or neurological disease, symptoms of rickets, or any other syndrome.

The control group comprised 64 healthy infants (32 male and 32 female) aged <1 year who were referred to the polyclinic with no specific symptoms. To discount any obstructive sleep respiratory events related to laryngomalacia, flexible laryngoscopy was performed for the infants of the control group and none were determined.

The flexible laryngoscopy was applied in the phoniatrics polyclinic (model 2850284, Karl-Storz, Tuttlingen, Germany). Each infant was held in an upright position and the flexible fiberoptic laryngoscope was placed over the larynx entering from the nose and passing within the oropharynx. The diagnosis of laryngomalacia was confirmed with supraglottic tissue collapse including the following conditions:

Mild laryngomalacia: Inspiratory stridor with no other symptoms or radiographic findings suggesting secondary airway lesion.
Moderate laryngomalacia: Cough, choking, regurgitation, feeding difficulty.
Severe laryngomalacia: Apnea, cyanosis, failure to thrive, pulmonary hypertension, cor pulmonale [14].

The video recordings of the patients with laryngomalacia were evaluated by two ear, nose, and throat physicians and the cases of laryngomalacia were classified (N.B., **Ö**.F.**Ç**).

Laboratory tests for all patients included calcium (Ca), phosphorus (P), parathormone (PTH), alanine transaminase (ALT), aspartate transaminase (AST), blood urea nitrogen (BUN), creatinine (Cre), and 25-hydroxy vitamin D (25-OH-D).

Blood samples taken from all infants into purple-capped tubes were centrifuged for 10 min at 4000 rpm and plasma was obtained. The measurement of 25-OH-D was determined in the obtained plasma with an HPLC device (UltiMate 3000, Thermo Fisher Scientific, Waltham, USA) using the Vitamin D ClinRep HPLC Kit (Recipe Chemicals Instruments, Munich, Germany).

Venous blood samples were obtained from the antecubital region by phlebotomy from the infants included in the study. Blood samples taken from all infants were centrifuged for 10 min at 4000 rpm and serum was obtained. The levels of Ca, P, ALT, AST, BUN, and Cre were measured using original kits with a biochemical autoanalyzer (Roche Diagnostics GmbH, Mannheim, Germany). Serum PTH levels were measured using original kits based on the electrochemiluminescence measurement principle with an autoanalyzer (Roche Diagnostics GmbH, Mannheim, Germany).

### 2.1. Statistical analysis

Data obtained in this study were analyzed statistically using IBM SPSS Statistics 22 for Windows (IBM Corp., Armonk, NY, USA). The conformity of the data to normal distribution was assessed with the Shapiro–Wilk test. In the comparison of two groups of variables with normal distribution, the independent samples t-test was applied. In the comparison of three or more groups, one-way ANOVA was used. The Tukey HSD test and Tamhane T2 test were applied as post hoc tests. For variables not showing normal distribution, the Mann–Whitney U test was applied for comparisons of two groups and the Kruskal–Wallis H test was used for three or more groups. Among the post hoc tests, the Dunn test was used. In the evaluation of categorical variables, the chi-square test and Fisher exact test were applied. Continuous variables were stated as mean ± standard deviation, median, and 25%–75% interquartile range values, and categorical variables were stated as number (n) and percentage (%). Values of p < 0.05 were accepted as statistically significant.

## 3. Results

Evaluations were performed for 64 infants with laryngomalacia, comprising 41 male (M) and 23 female (F) infants with a mean age of 4.6 ± 3.0 months, and for a control group of 64 healthy infants with a mean age of 4.5 ± 2.8 months. According to the laryngomalacia classification, 19 patients (8 F, 11 M) were in the mild laryngomalacia group, 26 patients (9 F, 17 M) were in the moderate laryngomalacia group, and 19 patients (6 F, 13 M) were in the severe laryngomalacia group. No statistically significant difference was determined between the groups with respect to the delivery route at birth, maternal milk, history of intensive care, or additional nutritional support (Table 1).

The 25-OH-D status was defined as moderate (≥30 ng/mL), insufficient (20–29 ng/mL), deficient (10–19 ng/mL), or severely deficient (<10 ng/mL). A statistically significant difference was determined in this distribution when the laryngomalacia patients and the control group were compared (p < 0.05) (Table 2).

In the comparison of biochemical data between the groups, no statistically significant difference was determined for Ca, AST, or Cre levels. A statistically significant difference was determined between the laryngomalacia patients and the control group with respect to 25-OH-D and PTH levels (p < 0.001). The mean 25-OH-D level was 20.60 ± 9.80 ng/mL in the laryngomalacia group and 29.90 ± 18.00 ng/mL in the control group. Statistically significant differences were determined between the groups with respect to BUN, P, and ALT values (p < 0.05) (Table 3).

**Figure 1:**
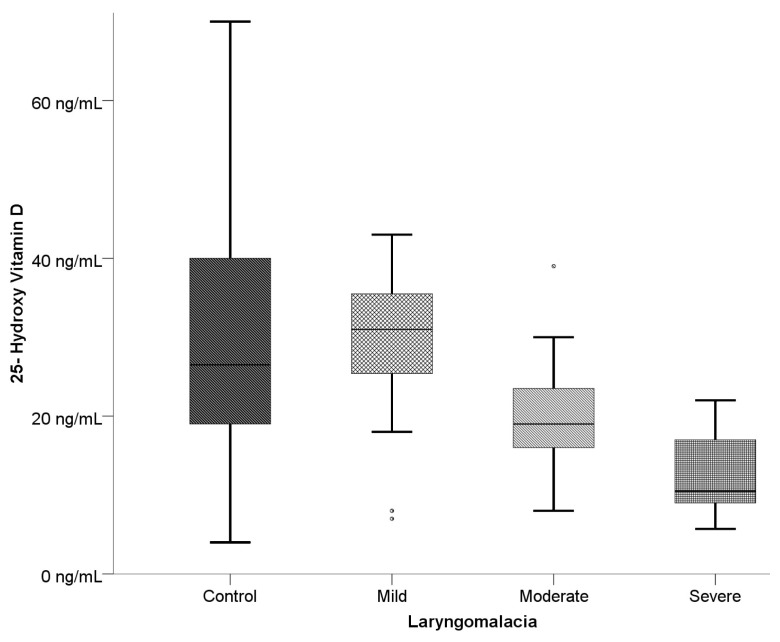
Box-plot curve of 25-OH-D levels according to the classification of laryngomalacia types.

**Figure 2:**
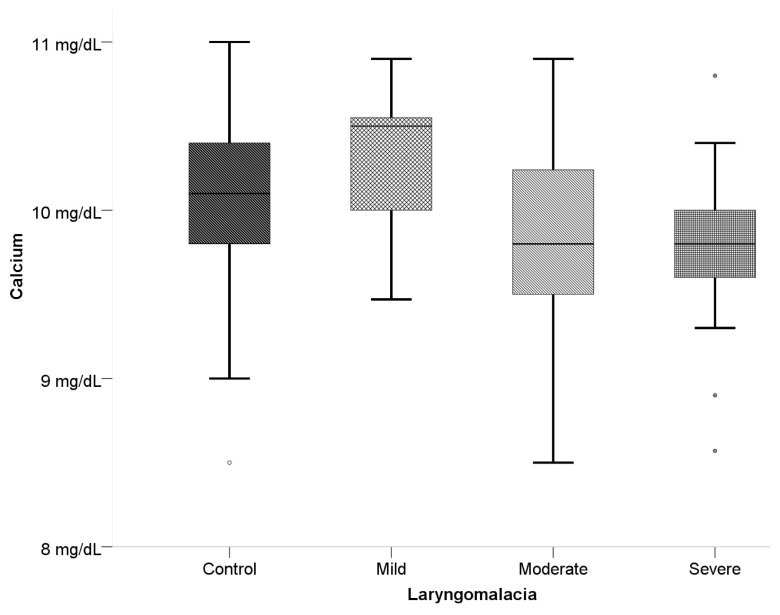
Box-plot curve of Ca levels according to the classification of laryngomalacia types.

When examined according to laryngomalacia type, statistically significant differences were determined between the groups for 25-OH-D, Ca, P, PTH, and ALT values. The 25-OH-D level was determined to be statistically significantly lower in the severe laryngomalacia group than in the mild and control groups (p < 0.001) (Table 4, Figures 1 and 2). The serum Ca level was statistically significantly higher in the mild group than in the moderate group and the severe group (p < 0.05). The increase was determined to be statistically significant in the mild and moderate laryngomalacia groups compared to the control group (p < 0.001). A statistically significant difference was determined between the moderate and severe laryngomalacia groups and the control group with respect to PTH levels (p < 0.001). The PTH level was statistically significantly lower in the severe group than in the control group and mild group (p < 0.001), and the level in the moderate group was lower than that in the control group.

## 4. Discussion

Laryngomalacia is a congenital disease and an important cause of neonatal stridor. Although the etiology is not fully known, there are several studies in the literature related to 25-OH-D levels and infant laryngomalacia [2,6–8].

In the literature, the 25-OH-D levels of children diagnosed with laryngomalacia have been shown to be lower than those of control group subjects [2,8]. Consistent with the literature, the mean 25-OH-D level of the current study’s patient group was found to be lower than that of the control group. To our knowledge, there is no study in the literature evaluating the relationship between the severity of laryngomalacia and 25-OH-D levels. The results of the current study showed that 25-OH-D levels were highest in the mild laryngomalacia group and lowest in the severe laryngomalacia group. Based on these data, it might be concluded that vitamin D deficiency increases the severity of laryngomalacia.

With the development of submucosal nerve hypertrophy, neurological dysfunction has been shown in severe laryngomalacia [15]. In another study, changes in the laryngeal tonus and sensorimotor integrative function of the larynx were found to be related to disease severity. This demonstrates that factors altering the peripheral and central pathways of the laryngeal adductor reflex have roles in the etiology of the signs and symptoms of laryngomalacia [14]. According to some studies, a low maternal vitamin D level can have a negative effect on the neuronal development of the fetus [11,12].

Retarded neuronal development or inflammation of the laryngeal structures may develop with vitamin D deficiency. This hypothesis was supported in a recent study that showed a high interleukin-6 level and vitamin D deficiency [8]. 1,25-Dihydroxy vitamin D, which functions as a steroid hormone, binds to a nuclear vitamin D receptor. These receptors are found at high levels in most inflammatory cells, such as dendritic cells, macrophages, and T and B lymphocyte cells [16]. This supports the theory that vitamin D could have a role in inflammatory and immune diseases. Inflammation of the larynx explains the dynamics of laryngomalacia with weak neuromuscular tonus [17].

In the current study, when vitamin D levels were classified as insufficient (20–29 ng/mL), deficient (10–19 ng/mL), and severely deficient (<10 ng/mL), vitamin D deficiency and severe deficiency were determined to be more prevalent in the laryngomalacia group than in the control group, and cases with insufficiency or a normal level were more common in the control group. This distribution demonstrates that the incidence of vitamin D deficiency is higher in patients with laryngomalacia. Many studies have been carried out to define vitamin D deficiency and insufficiency and to determine the normal range of 25-OH-D levels. In a study conducted with children between the ages of 0 and 12 years to define vitamin D deficiency and insufficiency and to determine the normal range of 25-OH-D levels, severe vitamin D deficiency was defined as a 25-OH-D level lower than 20 ng/mL, vitamin D deficiency as 21–29 ng/mL, and adequate levels as ≥30 ng/mL (with the preferred range being 40–60 ng/mL), while levels higher than 150 ng/mL were considered as indicating vitamin D intoxication [18]. In another study conducted with children and adolescents on the health of school-age children, rates of 15% for deficiency and 18% for sufficiency were found [19]. In a study by Çelik et al. [2], severe vitamin D deficiency was determined in laryngomalacia patients. In the current study, 25-OH-D, PTH, and BUN levels were observed to be lower in the laryngomalacia patient group than in the control group. The level of 25-OH-D was evaluated as 20.60 ± 9.80 ng/mL in the laryngomalacia patients and 29.90 ± 18.00 ng/mL in the control group. In the study by Çelik et al. [2], 25-OH-D was measured as 27.3 ± 12.4 ng/mL in the patient group and 37.7±13.1 ng/mL in the control group. The lower values in the current study may be attributed to the greater number of laryngomalacia cases and the levels of severity with the inclusion of a severe laryngomalacia group. Severe deficiency was found at a rate of 11% and deficiency was 23% in the laryngomalacia group according to vitamin D levels. In the control group, severe deficiency was 9% and deficiency was 9%. Rates of vitamin D deficiency and severe deficiency were thus higher in the laryngomalacia group than in the control group.

The measured levels of P and ALT were higher in the current study’s laryngomalacia patients than in the control group, and Çelik et al. [2] similarly reported significantly higher P levels in their laryngomalacia patient group. Physiologically, while vitamin D increases Ca and P absorption from the intestines, PTH causes an increase in active vitamin D for an increase in serum Ca as well as Ca absorption from the bones with inhibition of Ca excretion from the kidneys. PTH also provides phosphate excretion from the kidneys, or hypophosphatemia, and increases ALP activity [20,21]. The increasing PTH response to low vitamin D levels is important with respect to the Ca/P balance, and it causes demineralization to occur, as seen in cases of rickets and osteomalacia.

When laryngomalacia was classified according to types in the current study, significant differences were determined between the groups for Ca, P, PTH, and ALT. The levels of Ca, P, and PTH were observed to decrease when examined according to laryngomalacia type. In the mild laryngomalacia group, the Ca, P, and PTH levels were high, and the lowest levels were seen in the severe group. PTH should have been high in the metabolic pathway due to vitamin D deficiency. Therefore, the low level of PTH in the current study raises the question of whether hypoparathyroidism could be involved in the etiology of laryngomalacia independently of the vitamin D level.

There are some limitations to this study. Measurements of maternal vitamin D levels were not considered while designing the study. The saturations of the group with laryngomalacia were not measured, especially within the subgroup with severe laryngomalacia. Another limitation was that no evaluations were made after the treatment of the patients with low vitamin levels.

Previous studies have reported low vitamin D levels in laryngomalacia patients compared to control groups. In the current study, this deficiency was shown in detail. Unlike other studies, the link between vitamin D deficiency and increased severity of laryngomalacia has been shown. This study can be considered as a guide for further studies on this subject.

## 5. Conclusion

Vitamin D deficiency may have a role in the etiology of laryngomalacia. This view is supported by the results of this study, which has shown a decrease in vitamin D level associated with the classification of laryngomalacia. Further research into low PTH levels among infants with laryngomalacia may provide an explanation of the changes in Ca metabolism. It would be appropriate in future studies to investigate the response to vitamin D replacement therapy in patients with moderate and severe laryngomalacia.

## Figures and Tables

**Table 1:** Evaluation of the demographic data of the laryngomalacia patient group and the control group.

		Laryngomalacia	Control	p
Age, months, mean ± SD		4.60 ± 3.00	4.50 ± 2.80	0.804
Birth weight, g, mean ± SD		3062.30 ± 240.40	3066.50 ± 234.80	0.920
Sex	Male, n (%)	41 (64.1)	32 (50.0)	0.108
	Female, n (%)	23 (35.9)	32 (50.0)	
Route of delivery	Cesarean section, n (%)	41 (64.1)	39 (60.6)	0.715
	Normal spontaneous vaginal, n (%)	23 (35.9)	25 (39.4)	
Breast milk	−, n (%)	8 (12.5)	5 (7.8)	0.380
	+, n (%)	56 (87.5)	59 (92.2)	
Intensive care	−, n (%)	41 (64.1)	47 (73.5)	0.253
	+, n (%)	23 (35.9)	17 (26.5)	
Food consumption	−, n (%)	40 (62.5)	42 (65.6)	0.855
	+, n (%)	24 (37.5)	22 (34.4)	

Independent samples t-test; chi-square test; Fisher exact test; α: 0.05.

**Table 2:** Vitamin D levels of the laryngomalacia patient group and the control group.

	Vitamin D classification				
	Severe deficiency (0–9)	Deficiency (10–19)	Insufficiency (20–29)	Normal (≥30)	p
Laryngomalacia, n (%)	11 (17.2)	23 (35.9)	15 (23.4)	15 (23.4)	0.012*
Control, n (%)	9 (14.1)	9 (14.1)	17 (26.6)	29 (45.3)	

Chi-square test;

*: statistically significant difference in distribution.

**Table 3:** Evaluation of the 25-OH-D, Ca, P, PTH, ALT, AST, BUN, and Cre levels of the laryngomalacia patient group and the control group.

	Laryngomalacia	Control	p
25-OH-D, mean ± SD, ng/mL	20.60 ± 9.80	29.90 ± 18.00	<0.001*
Ca, median (Q1–Q3), mg/dL	10.00 (9.68–10.50)	10.10 (9.80–10.40)	0.415
P, mean ± SD, mg/dL	5.70 ± 0.73	5.41 ± 0.73	0.029*
PTH, median (Q1–Q3), pg/mL	21.0 (15.5–33.5)	31.5 (25.0–41.0)	<0.001*
ALT, median (Q1–Q3), U/L	21.5 (17.0–31.5)	16.0 (13.0–22.0)	0.001*
AST, median (Q1–Q3), U/L	38.0 (33.0–45.5)	36.0 (30.0–44.0)	0.355
BUN, median (Q1–Q3), mg/dL	7.0 (4.0–10.5)	8.5 (7.0–10.0)	0.045*
Cre, median (Q1–Q3), mg/dL	0.20 (0.14–0.34)	0.26 (0.22–0.32)	0.137

Mann–Whitney U test; independent samples t-test; α: 0.05;

*: statistical significance.

**Table 4:** Evaluation of 25-OH-D, Ca, P, PTH, ALT, AST, BUN, and Cre levels according to the classification of laryngomalacia types.

	Laryngomalacia classification				
	Control	Mild	Moderate	Severe	p
25-OH-D, median (Q1–Q3)	26.50 (19.00–40.00)^d^	31.00 (22.00–36.00)^d^	19.00 (16.00–24.00)	10.50 (9.00–17.00)^a^,^b^	p < 0.001*
Ca, median (Q1–Q3)	10.10 (9.80–10.40)	10.50 (10.00–10.60)^c^,^d^	9.80 (9.50–10.28)^b^	9.80 (960–1000)^b^	0.002*
P, mean ± SD	5.41 ± 0.73^b^,^d^	6.08 ± 0.48^a^,^c^	5.31 ± 0.74^b^,^d^	5.87 ± 0.68^a^,^c^	p < 0.001*
PTH, median (Q1–Q3)	31.50 (25.00–41.00)^c^,^d^	33.00 (15.00–45.00)^d^	19.00 (16.00–32.00)^a^	18.50 (14.00–23.00)^a^,^b^	p < 0.001*
ALT, median (Q1–Q3)	16.00 (13.00–22.00)^b^,^c^,^d^	26.00 (13.00–39.00)^a^	21.00 (18.00–31.00)^a^	22.50 (17.00–31.00)^a^	0.012*
AST, median (Q1–Q3)	36.00 (30.00–44.00)	37.00 (24.00–46.00)	38.00 (33.00–43.00)	40.50 (36.00–47.00)	0.222
BUN, median (Q1–Q3)	8.50 (7.00–10.00)	8.00 (4.00–14.00)	6.80 (4.30–10.00)	6.75 (4.00–9.00)	0.189
Cre, median (Q1–Q3)	0.26 (0.22–0.32)	0.21 (0.10–0.40)	0.20 (0.16–0.32)	0.20 (0.10–0.30)	0.454

One-way ANOVA; post hoc: Tukey HSD test; Dunnett test; Kruskal–Wallis H test; post hoc: Dunn test; α: 0.05;

*: statistical significance;

a: significant difference compared to control group;

b: significant difference compared to mild group;

c: significant difference compared to moderate group;

d: significant difference compared to severe group.
